# Reduced mrna expression level of corticotropin-releasing hormone-binding protein is associated with aggressive human kidney cancer

**DOI:** 10.1186/1471-2407-13-199

**Published:** 2013-04-22

**Authors:** Hossein Tezval, Faranaz Atschekzei, Inga Peters, Sandra Waalkes, Jörg Hennenlotter, Arnulf Stenzl, Jan U Becker, Axel S Merseburger, Markus A Kuczyk, Jürgen Serth

**Affiliations:** 1 Department of Urology and Urological Oncology, Hannover Medical School, Hannover, Germany; 2 Department of Urology, Eberhard Karls University of Tuebingen, Tuebingen, Germany; 3 Institute of Pathology, Hannover Medical School, Hannover, Germany

## Abstract

**Background:**

Significance of Urocortin (Ucn or UcnI), Ucn2, Ucn3 and their receptors, Corticotropin Releasing Factor Receptor 1 and 2 (CRFR1 and CRFR2), and the binding protein, Corticotropin-Releasing Hormone-Binding Protein (CRHBP) in oncology is growing rapidly. The objective of our study was to assess the expression of the CRHBP mRNA and protein in renal cancer.

**Methods:**

Tumoral tissues of 78 patients with clear cell renal cell cancer and their corresponding normal tissues were analyzed using quantitative mRNA expression analysis for detection of mRNA expression level. Protein expression and tissue localization of CRHBP protein in renal specimens was evaluated using western blotting, immunohistochemistry and double immunofluorescence, respectively.

**Results:**

We found an approx. 33 fold decrease of average CRHBP mRNA level in tumoral tissues compared to paired normal tissues (p<0.001). Diminished CRHBP mRNA expression was positively correlated with advanced, metastasized and higher stage of disease (*p*<0.001, *p*=0.026, *p*=0.028 respectively). CRHBP protein was detected in glomeruli and proximal tubules of normal kidney while none or weak immunopositivity was found in cc-RCC (p<0.001).

**Conclusions:**

The expression analysis of CRHBP shows that cc-RCC is characterized by a significant loss of CRHBP mRNA expression that furthermore is associated with a more aggressive state of tumors. Depletion of CRHBP proteins also indicate that the protein as part of the UCN system may be involved in renal carcinogenesis.

## Background

The Corticotropin releasing factor (CRF) system in human includes all naturally occurring CRF peptide analogues namely Urocortin (Ucn) and Urocortin 3 (Ucn3), known as CRF counterparts in the periphery, the CRF receptors 1 and 2 (CRFR1 and CRFR2), and finally Corticotropin-releasing hormone-binding protein (CRHBP) [1-4]. The existence and translation of Urocortin 2 (Ucn2) in human is still unclear. It has been shown that CRF analogues can inhibit tumor progression, can modulate proliferation and apoptosis, and can hinder angiogenesis by reduction of VEGF expression *in vivo*, through activation of CRF receptors, especially CRFR2 in different tumor entities [5-8]. Expression and the pathophysiological relevance of the CRF system has been reviewed for different human cancers [9,10]. Recently, we reported the expression of Ucn and CRFR2 in clear cell renal cell carcinoma (cc-RCC) [11]. In our study, a nuclear migration of Ucn and loss of expression of vascular CRFR2 in cc-RCC could be demonstrated [11]. Expression of CRHBP on mRNA level has been reported in human normal kidney [12] but there is still no data available about the expression of CRHBP in kidney cancer. Moreover, for other tumor entities it has been pointed out that expression patterns of the CRF system are related to grade and stage of tumors [10].

To assess a potential relevance of CRHBP expression alterations for cc-RCC we first compared the mRNA expression levels of CRHBP in cc-RCC fresh frozen specimens and paired normal appearing tissue samples using quantitative RT-PCR analysis. Moreover, relative mRNA expression levels were statistically evaluated for association with clinicopathological parameters of cc-RCC patients. Presence and localization of CRHBP protein expression in normal and malignant kidney tissues were investigated using immunohistochemistry and immunofluorescence.

## Methods

### Patients’ characteristics

The present study included sample cohorts both of fresh frozen and paraffin embedded tissues. Fresh frozen samples of tumors and a subset of corresponding tumor free tissues were obtained from 109 patients subjected to kidney surgery between 2001 and 2005 in the Eberhard Karls University Tuebingen. Tissue preparation, storage, pathological evaluation, tumor stage assessment according to the UICC 2002, nuclear grading, and data management have been previously described [13]. The ethical committee of the institution (the Eberhard Karls University Tuebingen) approved the study and informed consent of patients was obtained. The study was carried out in compliance with the Helsinki Declaration. For mRNA expression analysis we selected fresh frozen specimens of 78 tumors with the histological subtype of cc-RCC and available paired normal appearing tissue samples. Organ-confined RCC was defined as pT≤2 and N0/M0 and advanced as pT ≥ 3 and/or N+/M+. Clinical and histopathological data of this group are summarized in Table 1.

**Table 1:** Clinical and histopathological data of patients with renal cell cancer used for qPCR

**Clinico-pathological parameters**	**Number of patients**	**%**
Total	109	100
Age (mean; ± Standard deviation)	63 ± 11.9	
male	70	64.2
female	39	35.8
Side		
left	49	45
right	60	55
Surgery		
Partial nephrectomy	27	24.8
Radical nephrectomy	82	75.2
Histological subtypes		
clear cell	78	71.6
papillary	22	20.2
chromophobe	2	1.8
other/ not classified	7	6.4
pT1a	34	31.2
pT1b	30	27.5
pT2	5	4.6
pT3a	13	11.9
pT3b/c	27	24.8
pT4	0	0
Synchronous Lymph nodes metastasis	11	10.1
Synchronous distant metastasis	23	21.1
Advanced disease (pT3-4 and/or N/M+)	48	44.0
G1	18	16.5
G1-2	15	13.8
G2	58	53.2
G2-3	7	6.4
G3	11	10.1

Paraffine embedded tissue samples of tumor, invasion front and adjacent histopathologically normal tissues were obtained from an independent group of patients, subjected to nephrectomy and arranged as tissue microarrays as described before [13,14]. Clinical and histopathological data of the subset of 33 patients with cc-RCC considered for immunhistochemical (n=16 patients) or immunofluorescence (n=17 patients) analysis of paraffin embedded tissue microarray specimens are shown in Table 2.

**Table 2:** Clinical and histopathological data of paraffine embedded tissue samples of renal cell cancer cases

**Variable**		**All patients**^1^	**IHC-TMA**^**1**^	**IF-TMA**^**1**^
		**n (%)**	**n (%)**	**n (%)**
**Total**		33 (100)	16 (48)	17 (52)
**Gender**	Female	13 (39)	7 (43)	6 (35)
	Male	20 (61)	9 (57)	11 (65)
**Age (mean)**		59.03	61.7	56
**pT stage**	T1	0 (0)	0 (0)	0 (0)
	T2	16 (48)	7 (43)	9 (53)
	T3	15 (45)	9 (57)	6 (35)
	T4	2 (6)	0 (0)	2 (12)
**Lymph node**	N0	12 (36)	4 (25)	8 (47)
	N1	3 (9)	3 (18)	0 (0)
	Nx	18 (55)	9 (56)	9 (53)
**Metastasis**	M0	23 (69)	11 (69)	12 (70)
	M1	8 (24)	4 (25)	4 (24)
	Mx	2 (7)	1 (6)	1 (6)
**Tumor grade**	G1	4 (12)	4 (25)	0 (0)
	G2	25 (76)	10 (62)	15 (88)
	G3	3 (9)	1 (6)	2 (12)
	G4	0 (0)	0 (0)	0 (0)
	Unknown	1 (3)	1 (6)	0 (0)

^1^subgroup with clear cell histology; IHC, immunohistochemical analysis; IF, immunofluorescence analysis; TMA, tissue microarray analysis.

### Nucleic acid extraction and quantitative real time PCR

RNA extraction from the fresh frozen tissue group and cDNA synthesis were performed as described before [15]. Briefly, quantitative real time RT-PCR analyses were performed in duplicate with an ABI 7900 Fast Sequence Detection System using TaqMan gene expressionn assays and universal PCR master mix according to the manufacturer's specifications (life technologies) [15]. The TaqMan assays used were *CRHBP* (Assay ID: Hs00181810_m1), *GUSB* (Assay ID: Hs00939627_m1), *RPL13A* (Assay ID: Hs03043885_g1) and *HPRT1* (Assay ID: Hs99999909_m1). The human *GUSB*, *RPL13A* and *HPRT1* transcripts served as endogenous controls. Additional no-template, no reverse transcription and blank controls were included in each run.

Relative quantities of transcripts were calculated using the SDS 2.3 Manager, data assist v2.0 Software and the delta-delta Ct method [16,17]. The reference Ct values both for CRHBP and the endogenous controls were calculated from the whole tissue sample group and applied as a surrogate biological control for computation of relative quantities.

### Western blot analysis

Western blotting was performed according to standard protocols. Briefly, blots were incubated with primary goat antihuman antibody for CRHBP (1:1000 dilutions, AF2796, R&D systems GmbH, Wiesbaden-Nordenstadt, Germany) and biotinylated horse anti-goat IgG Antibody (1:200 dilutions, BA 9500, Vector, enzo life sciences GmbH, Lörrach, Germany). For detection of the loading control we used mouse monoclonal anti beta Tubulin (1:1000 dilution, DSHB, Iowa, US) as primary and peroxidase labeled antimouse antibody as secondary antibody (1:10000 dilution, NIF 825, Amersham, GeHealthcare, Freiburg, Germany). Antibody-protein complexes were visualized using a super west dura kit (Thermo scientific, 34076) and Amersham Hyperfilm (Ge Healthcare) following scanning of the film.

### Immunohistochemical and immunofluorescence analyses

Immunohistochemical (IH) and immunofluorescence (IF) analyses of tissue microarrays were carried out as described before [11,13,18]. For IF analysis, anti-human CRHBP, a goat polyclonal antibody (1:100 dilutions, AF2796, R&D systems GmbH, Wiesbaden-Nordenstadt, Germany) and secondary antibody as described above for western blotting was applied. Rabbit anti-human MUC-1 polyclonal antibody (1:100 dilutions, ab15481, abcam, Cambridge, UK) and rabbit polyclonal anti-human nephrin (1:100 dilutions, ab58968, abcam, Cambridge, UK) were used for double IF staining for specific detection of distal tubuli (Muc-1) and glomeruli (Nephrin) [19,20]. As secondary antibody we used biotinylated anti mouse-anti rabbit (1:200 dilutions, Vector, BA 1400, enzo life sciences GmbH, Lörrach, Germany). The paraffin embedded tissue sections were demasked and stained following Avidin/Biotin blocking (Vector Laboratories, Burlingame, CA) by the use of ABC and tyramide based ATTO-488 and ATTO-655 fluorescent dyes as specified before [11,18]. A negative control was included using omitting the primary antibody.

### Statistical analysis

For comparison of kidney tumor tissues and paired tumor adjacent normal tissue samples the paired t-test was applied for evaluation of relative mRNA quantitation results while the NcNemar Chi - square test was used for nonparametric pairwise comparison of immunostaining results. For the immunohistochemically stained tissue microarray only signals in normal tubular epithelial or tumor cells were considered. Tissue samples from the immunofluorescence stained tissue microarray were evaluated for the overall intensity of CRHBP related fluorescence detected within the field of view independent from morphological informations of DAPI staining of nuclei. Univariate logistic regression models were carried out for independent group comparisons of measured mRNA levels as described before [15]. Means and standard deviations (sd) per group, odds ratios (OR), corresponding 95% confidence intervals (CI) and two-sided p-values are presented. P ≤ 0.05 was considered to be statistically significant.

## Results

### Analysis of mRNA expression of CRHBP in normal kidney and kidney cancer

**Figure 1:**
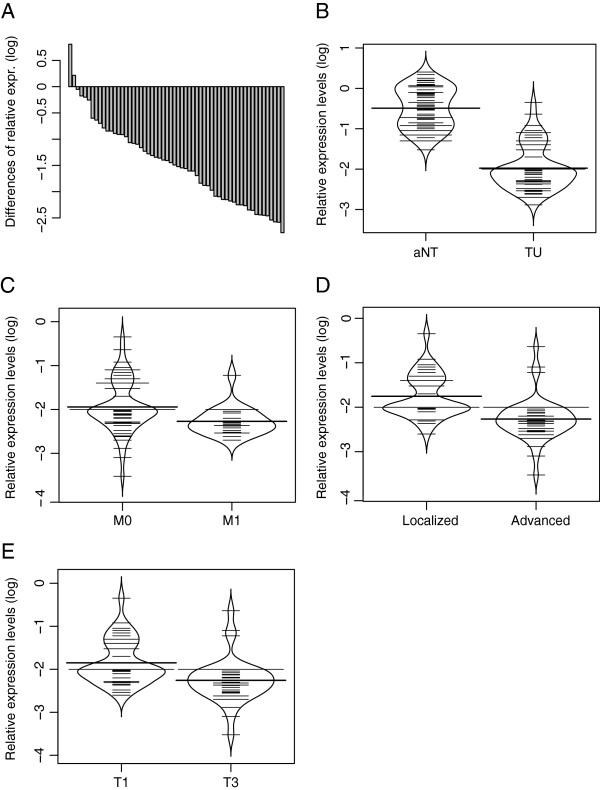
**Relative CRHBP mRNA expression levels in renal tissues and comparison with clinicopathological parameters. A**) Assorted difference plot for illustration of relative mRNA expression level differences between tumour and paired adjacent normal tissue samples of cc-RCCs. **B**) Groupwise bean plot comparison of relative CRHBP mRNA expression level in cc-RCC (TU) and normal kidney (aNT)shows approx. 33 fold reduction in CRHBP mRNA level (p<0.001). **C**) CRHBP mRNA level comparison in M+ and M- in cc-RCC indicates a significant reduction of CRHBP in metastasized state of the disease, *p*=0.026. **D**) CRHBP mRNA level comparison in advanced and localized disease demonstrates a higher expression of CRHBP in localized cc-RCC, *p*<0.001. **E**) CRHBP mRNA level comparison in high (T3) and low stage (T1) tumors (cc-RCC) shows a significant reduction of CRHBP in higher stages of disease, *p*=0.028.

Using 5´ exonuclease fluorogenic real-time PCR assays (qPCR) for quantitative expression analysis of CRHBP mRNA levels, we found in pairwise comparisons in most of cases a loss of expression in tumor tissues as indicated by the negative differences of sorted pairwise relative expressions in tumor and normal tissue (Figure 1A). Group comparison of tumors (clear cell carcinoma subtype (n=78)) and paired normal tissue samples showed a mean relative expression of 0.0091 and 0.334 respectively (Figure 1B) corresponding to a 33 fold reduction for the mean relative mRNA levels of CRHPB in tumor tissues. Statistical analysis using the paired t-test confirmed that means of both groups are different (*p* <0.001).

### Analysis of CRHBP protein expression and tissue localization in kidney tissues

**Figure 2:**
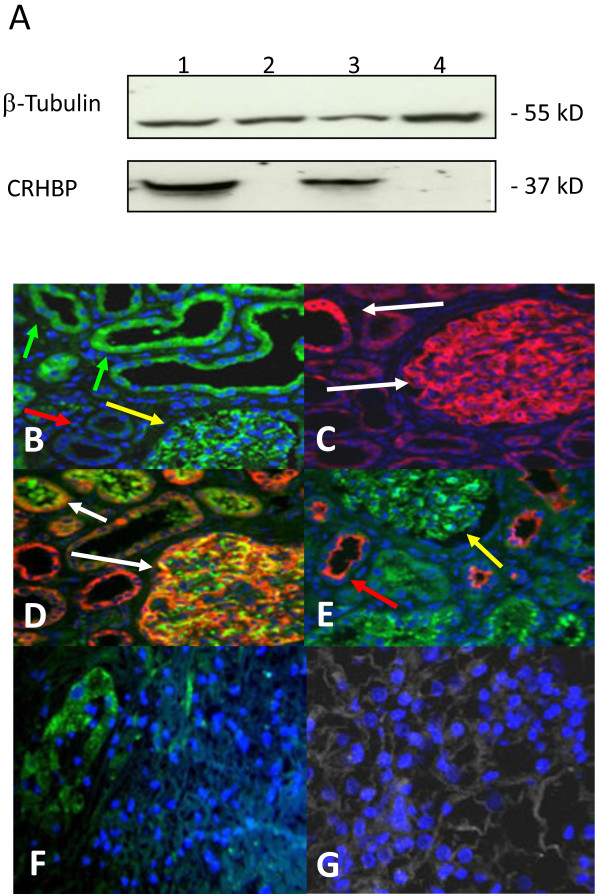
**CRHBP protein expression and tissue localization in cc-RCC and normal kidney. A**) Western blotting shows a tumor specific loss of CRHBP protein in lane 2 and 4. Positive signals for CRHBP were observed in most of the normal specimens. Detection of β-Tubulin has been used as an internal loading control. **B**) CRHBP immuno-signalling in proximal tubuli (green arrows), glomeruli (yellow arrows) and lack of immunopositivity in distal tubules of normal kidney (red arrows). **C**) Immunopositivity for nephrin detected in glomeruli and proximal tubules in normal kidney. **D**) Double immunofluorescence staining of nephrin (red) and CRHBP (green) demonstrating co-expression in glomeruli and proximal tubules of normal renal tissues. **E**) Double immunofluorescence staining of CRHBP (green) and distal marker protein MUC-1 (red) in normal kidney **F**) Invasion front tissue demonstrating reduction of CRHBP protein immunosignaling when normal (left side) and tumor tissue areas (right side) are compared **G**) CRHBP immunofluorescence staining (green) with nuclear counterstaining using Dapi (blue) in tumor tissue of cc-RCC.

To characterize the specificity of the CRHBP antibody we first carried out western blot analysis in test lysates of four pairs of cc-RCC tumors and corresponding normal fresh frozen tissues. As a result we obtained a single band of expected molecular weight of 37 kD for each of the normal tissues as exemplarily shown in Figure 2A (lanes 1 and 3) for two tissue pairs thus indicating the specificity of the antibody used. None of the four tumors exhibited a detectable signal in the range of the molecular weight of CRHBP (Figure 2A, lanes 2 and 4).

Next we used immunofluorescence analysis of a tissue microarray representing 17 cc-RCC to characterize the distribution of immunopositivity of CRHBP in normal and tumor tissues. Considering that a previous study detected mRNA expression both in glomeruli and podocytes of normal tissues, we also exemplified costaining of CRHBP both with the anti-nephrin antibody for detection of glomeruli and/or podocytes [20] as well as the anti-MUC-1 antibody as a marker for distal tubules in seven paraffin sections of cc-RCC independent from the tissue microarray samples [19]. As a result we found CRHBP immunopositivity in glomeruli and podocytes, as well as intensive signals in MUC1-1 negative tubular structures of normal appearing tissues (Figure 2B-E). In contrast none or faint signals were observed in tumor tissues (Figure 2G). To assign immunopositivity to morphologically defined areas within tissue samples we also used normal immunohistochemistry for staining of another tissue microarray consisting of each 16 tumor, invasion front and paired normal samples from cc-RCC. Tissue specimens showed high immunopositivity located in tubules of normal tissue areas but low or lacking positivity in the tumor areas as examplary demonstrated for an invasion front sample in Figure 2F.

Comparison of immunopositivity in tumor and paired normal tissues could be carried out in 15 out of 16 and 15 out of 17 cases analysed by immunohistochemistry or immunofluorescence, respectively. The comparison revealed that 15 out of 15 (100%, immunohistochemically stained tissue microarray) and 13 out 15 (87%, immunofluorescence analysis) tissue pairs with cc-RCC histology in tumors demonstrated clearly higher immunopositivity in normal tissues.

Two tissue pairs were either detected negative or showed comparable immunofluorescence signals in tumor and normal tissues. Statistical comparison of differences demonstrated a significant difference in immunopositivity between tumor and paired normal tissues in both microarrays (p<0.001, p<0.001, McNemar chi-square analysis). Considering that only one tumor tissue out of 28 was detected to exhibit immunopositivity, analysis of further tissue microarrays for detection of a possible association of tumoral immunopositivity with clinicopathological parameters was not carried out.

### Clinicopathological relevance of relative CRHBP mRNA expression levels in cc-RCC

In contrast to the detection of CRHBP immunopositivity, mRNA levels in tumor tissue could be differentiated. We therefore statistically compared relative mRNA expression levels with histology and clinicopathological parameters of tumor patients (Figure 1). Our statistical evaluation of CRHBP mRNA levels within the tumor group revealed that reduced CRHBP levels are associated with advanced, metastasized and higher stages of disease (*p*<0.001, *p*=0.026, *p*=0.028 respectively, Figure 1C-E).

## Discussion

Here we demonstrate that CRHBP expression is depleted both on mRNA and most likely also on protein level in cc-RCC. Moreover, we found that loss of CRHBP mRNA expression is correlated with advanced, metastasized and higher stages of disease. To our knowledge, tumor specific reduction of CRHBP expression has not been reported in any human malignancy so far, although results indicative of a decreased expression of corticotropin-releasing hormone-binding protein mRNA in pituitary adenomas have been described before [21]. On the other hand, various studies show that Ucn, CRFR1 and CRFR2 are differently regulated and expressed in human cancer [10,11]. Moreover, several i*n vitro* and *in vivo* studies reported that activation of CRFR2 suppresses the neovascularization through reduction of VEGF production. The loss of function, blocking or gene knock out of CRFR2 has been found to cause an up-regulation of VEGF in turn inducing neovascularization [5,7,8]. This is in good concordance with our previous finding that the CRFR2 protein is not expressed in vessels of cc-RCC [11], a cancer that is characterized by increased VEGF levels and substantial neovascularization. CRHBP is the only example of a neuro-peptide-binding protein that circulates in the plasma. CRHBP binds the Corticotropin –Releasing factor (CRF) and other CRF-family peptides, e.g. Urocortin (Ucn) with a high affinity but does not interact with Ucn3 [22,23]. It has been hypothesized that extracellular CRHBP may serve as a ligand trap thus potentially be involved in regulation of the bound/free ligand equilibrium of locally required peptides and representing a potential pharmacological therapeutic target [24,25]. Considering that ligand concentrations of locally required peptides have been shown to affect proliferation and angiogenesis of cancer cells, we suppose that the reduction of CRHBP mRNA and protein expression in tumors also contribute in those pivotal tumorigenic processes.

Immunopositivity was detected mainly in proximal tubular epithelial cells of normal tissue for both CRHBP itself in the present study as well as for the CRHBP ligand Ucn in a previous analysis [11], hence also supporting the hypothesis that CRHBP is involved in tumor specific alterations of the UCN system. Finally this point of view, is strengthened by our statistical findings revealing a significant relationship between reduced tumoral mRNA expression levels and the state of more aggressive tumors in cc-RCC. Considering that previous analyses support the relevance of the UCN system for human carcinogenesis including also renal cancer, we hypothesize that alteration of CRHBP as a putative modulator of UCN levels may also contribute to the development and course of cc-RCC.

Although we found reduction of both, mRNA levels and immunopositivity on protein levels in cc-RCC tissue compared to normal kidney, this study obviously does not allow functional implications about the role of CRHBP in cc-RCC. While a direct head to head association between mRNA and protein levels has not been shown in our study, it is known that epigenetic changes including altered expression of micro- or long non-coding RNA may effect protein expression after transcription. Such a candidate epigenetic effector is miR-200bc/429 which has already been annotated to the CRHBP gene (UCSC human genome browser) [26]. Therefore functional analyses such as targeted knock out of CRHBP gene in a cc-RCC tumor model or epigenetic characterization of the UCN system should provide an improved understanding of the CRHBP contribution to the pathogenesis of cc-RCC.

Interestingly, Ucn family peptides have also been detected in human circulation and urine [4,27] while CRHBP expression beside those observed in normal tubules of the kidney is also found in podocytes of renal glomeruli. Whether Ucn and CRHBP levels in blood and urine become altered and possibly affect renal cancer progression or can serve as biomarker therefore appear as further relevant questions remaining to be clarified in future studies.

## Conclusion

In conclusion our data give evidence that altered CRHBP expression as part of the UCN system is involved in kidney cancer. Our results underline the potential biological relevance of the CRF peptide family for renal cancer and may also be of clinical relevance for future diagnostic or molecular therapeutic approaches.

## Abbreviations

CC-RCC: Clear Cell Renal Cell Carcinoma; CI: Confidence Interval; CRHBP: Corticotropin-Releasing Hormone-Binding Protein; CRF: Corticotropin-Releasing Factor; CRFR1: Corticotropin-Releasing Factor Receptor 1; CRFR2: Corticotropin-Releasing Factor Receptor 2; OR: Odds ratio; qRT-PCR: Quantitative Real Time PCR; Ucn: Urocortin; Ucn3: Urocortin 3.

## Competing interest

The authors declare that they have no competing interests.

## Authors’ contributions

HT wrote the manuscript and designed the study as director the study group. FA performed the q-PCR. IP and SW helped by interpretation the data and design. JH and AS provided the tissue material and analysed the data bank. JUB performed the pathological findings. ASM and MAK helped by design and correction the Manuscript. JS performed the WB analysis and helped by statistical analysis. All authors read and approved the final manuscript.

## Pre-publication history

The pre-publication history for this paper can be accessed here:

http://www.biomedcentral.com/1471-2407/13/199/prepub
